# Oestrogen shuts the door on SOX9

**DOI:** 10.1186/1741-7007-8-110

**Published:** 2010-08-31

**Authors:** Lindsey Mork, Blanche Capel

**Affiliations:** 1 Department of Cell Biology, Duke University Medical Center, Durham, NC, USA

## Abstract

Oestrogen exerts a robust yet imperfectly understood effect on sexual development in vertebrate embryos. New work by Pask and colleagues in BMC Biology indicates that it may interfere with male development by preventing nuclear localization of SOX9, a master regulator of the testis differentiation pathway.

See research article http://www.biomedcentral.com/1741-7007/8/113

## 

Vertebrates exposed to exogenous oestrogens during embryonic development often exhibit extensive sexual abnormalities as adults, ranging from genital and reproductive tract malformations to infertility or full male-to-female sex reversal. Although these effects have been studied at the phenomenological level for decades, no consensus has emerged regarding the molecular details of how this hormone influences primary sex determination, the process in which the initially bipotential gonads commit to testis or ovary differentiation. In the present issue of *BMC Biology*, Pask *et al. *[1] show that in marsupials, ectopic oestrogen can alter the subcellular localisation of SOX9, a highly conserved member of the testis differentiation pathway, providing a potential mechanism for oestrogen's feminising influence.

Non-mammalian vertebrate embryos are particularly sensitive to oestrogen-induced male-to-female sex reversal and reproductive dysgenesis. Suppression of oestrogen synthesis in these animals has the opposite effect, causing predetermined females to develop as males. These findings collectively indicate that oestrogen is necessary and sufficient for female development in most non-mammalian vertebrates. While this may be an advantageous system under some circumstances, the accumulation of oestrogenic chemicals as a result of agricultural runoff and pollution from modern pharmaceutical and industrial practices can have grave consequences on sexual development in egg-laying species, especially those that lay their eggs near water.

Mammalian viviparity provides some insulation from the potential hazards of the environment. The mammalian placenta can shield the developing embryo from exogenous hormones through its ability to convert and deactivate steroids. In contrast to egg-laying species, eutherian mammals have evolved a genetic (hormone-independent) system of sex determination based on the presence or absence of the Y chromosome. XY embryos express a gene on the Y chromosome, *Sry*, that directs the bipotential gonads to develop as testes (reviewed in [2]). XX embryos, which do not carry *Sry*, develop ovaries in an oestrogen-independent manner. This system does not seem to be influenced by exogenous oestrogen. Developing rodents treated *in utero *with synthetic oestrogenic compounds at clinically relevant doses showed no defect in primary sex determination (e.g. [3]), although ovotestes were infrequently observed in animals exposed to high doses [4]. Convincing genetic evidence that oestrogen does not play a role in the initial decision to develop as a female mammal came from studies of mice carrying null mutations of the oestrogen receptors or the aromatase gene, which encodes the enzyme that produces oestrogen (reviewed in [5]). In these mutants, foetal ovary development occurred normally.

However, the case is somewhat different in marsupials. Marsupial development is divided into a short period of placental gestation followed by a long period of postnatal development during lactation. Sex determination in this group of mammals also depends on the XY-SRY system, but the process occurs around the time of parturition, not mid-gestation as in eutherians (reviewed in [6]). Marsupial young are therefore susceptible to environmental influences during the sex-determining period. Interestingly, marsupials have maintained the sensitivity to oestrogen exposure that was lost in the eutherian lineage, such that XY pups that are fed estradiol with their mother's milk exhibit disrupted testis differentiation or even full male-to-female sex reversal if the pups are born and treated one day premature [6].

The paper by Pask *et al. *[1] provides a cellular explanation for the sensitivity of the marsupial gonad to oestrogen. Using the tammar wallaby (Figure 1) as a model marsupial, they first determined the gene expression patterns of eight genes known to play important roles in eutherian gonad development during a window extending from three days before birth to ten days post partum, which covers the bipotential stage as well as the onset of testis or ovary differentiation. They then compared the levels of these genes in XY gonads that were explanted one day before birth and cultured for five days in the presence of oestrogen or control medium. All but one of the 'male' genes examined showed dramatic down-regulation upon oestrogen treatment, in accord with the male-to-female morphological sex reversal observed in these samples. However, the authors were surprised to observe that a fourth gene, *SOX9*, was not down-regulated in the oestrogen-treated samples. *SOX9 *(SRY-box containing gene 9) is a highly conserved HMG-box transcription factor that in mice is directly activated by SRY and can induce full female-to-male sex reversal when misexpressed in XX embryos (reviewed in [2]). Its functional significance for testis development in species other than mouse and human has not yet been demonstrated because of the difficulty of performing genetic manipulations in non-model systems, but it is expressed in the testis cords (and in some cases during the bipotential period) of all vertebrate species examined to date [7].

**Figure 1:**
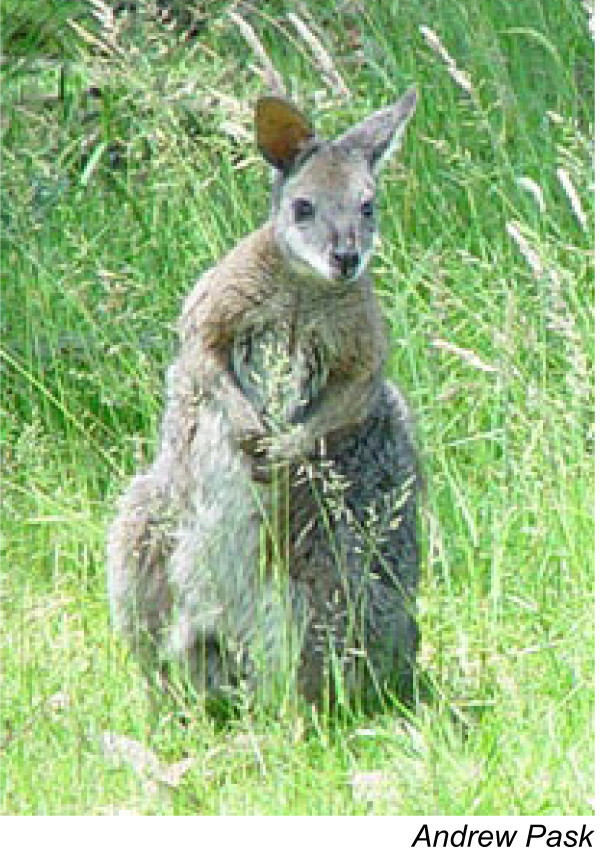
**A tammar wallaby (*Macropus eugenii*)**.

When Pask *et al. *examined SOX9 protein localisation in the oestrogen-treated marsupial gonads, they saw that the protein was indeed still present, but diffuse and cytoplasmic, in sharp contrast to its normal dense nuclear pattern (Figure 2). They concluded that oestrogen signalling may cause SOX9 to be shuttled out of the nucleus, preventing it from activating testis development, and thus allowing ovarian differentiation to proceed. Assuming that SOX9 does indeed play an important functional role during testis development in non-eutherian species, this effect could explain how oestrogen can exert such a potent sex-reversing effect on developing gonads.

**Figure 2:**
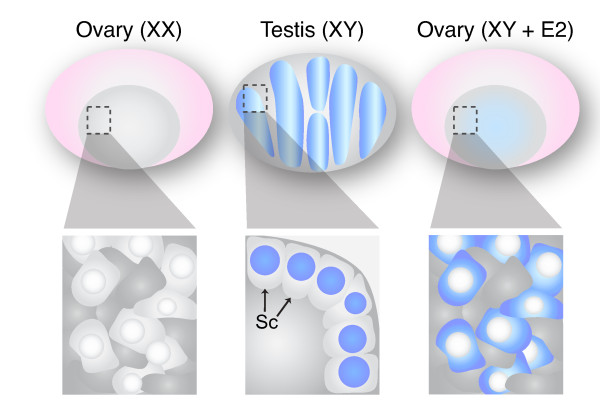
**In tammar pouch young, the critical male gene SOX9 (blue) is not expressed in differentiating ovaries but is highly enriched in Sertoli cell (Sc) nuclei within testis cords**. Perinatal treatment of XY young with β-estradiol resulted in cytoplasmic localization of SOX9 and ovarian development.

Regulation of SOX9 protein localisation in mice has received a fair amount of attention in recent years, primarily because a dramatic shift from cytoplasmic to nuclear localisation occurs upon activation of the male pathway in the murine XY gonad. SOX9 localisation can be regulated by prostaglandin signalling, importin-β1, calmodulin, and sumoylation/ubiquitination (reviewed in [8]). Oestrogen may act through one of these pathways to block SOX9 nuclear entry in marsupial gonads.

In the red-eared slider turtle, SOX9 is initially expressed at high levels during the bipotential period in both presumptive sexes, then declines precipitously at the onset of ovarian differentiation [9]. Treatment of turtle eggs with oestrogen during the bipotential period led to suppression of SOX9 at the transcriptional level; however, the protein remained nuclear in the few cells that still expressed it [9]. In addition, oestrogen receptor alpha was recently shown to work in conjunction with the transcription factor FOXL2 to block *Sox9 *transcription in the adult mouse ovary [10]. These findings indicate that the repressive effect of oestrogen on SOX9 may rely on different underlying mechanisms in different vertebrate species or at different stages of development.

Although sex determination in marsupials retains the ancestral sensitivity to oestrogen, it is not clear that oestrogen is involved in the endogenous process of making an ovary in these animals. The β-estradiol content of developing tammar gonads is minimal [6], and no attempts to inhibit aromatase activity in pouch young have been reported to date. Nevertheless, the line of work pursued by Pask and colleagues reveals a new role for oestrogen in the regulation of SOX9 protein subcellular localisation and addresses the critical question of exactly how environmental oestrogens affect sex determination in sensitive vertebrate species.
